# War against Bogus Colleges and Degrees

**Published:** 1899-01

**Authors:** 


### EDITORIAL.

### War Against Bogus Colleges and Degrees.

The Illinois Teachers' Association held a meeting a few days ago at which some definite action was taken upon the subject of the above heading.

The fact that in the State of Illinois there exists quite a number of so-called colleges whose business is not education, but consists chiefly of issuing and selling to whomsoever may buy, diplomas of various kinds, these purporting to come from legally organized institutions. There were present a large number of college presidents and instructors.

President Rogers, of Northwestern University, said "Illinois has disgraced the United States more than any other State in the Union. The offenders are chiefly medical and law schools. There are seventeen fraudulent degree-conferring shops in Chicago to-day. There were seventeen yesterday, said President Harper; there are probably more to-day.

There is one collegiate institution in the State which has one instructor. He confers the Bachelors degree, the Masters degree, the Doctors degree and the degree of Doctor of Divinity. He has been the subject of legislation on the part of the British Parliament."

After due consideration this association decided to petition the Legislature for the establishment by law of a body to be known as the Educational Commission, to be composed of not less than six nor more than nine members, to be appointed by the Governor and confirmed by the Senate, appointees to be members of the faculties or Boards of Trustees or other governing bodies of educational institutions within the State.

The eommission is to be vested with power to grant and deprive institutions of the degree-conferring rights. The committee to secure such legislation is composed of President H. W. Rogers, Northwestern College; President W. R. Harper, of Chicago University; President A. S. Draper, of Illinois State University; President J. H. Findlay, of Knox College; President J. E. Bradley, Illinois College; President C. A. Blanchard, Wheaton College; President E. M. Smith, Illinois Wesleyan College; President De Blois, Shurtleff College; President Ruthrauth Carthage; President Turner, Lincoln College; President W. R. Bridgman, Lake Forest University.

In discussion of the subject President Rogers said educational institutions in this State and the same is true of other States, must now be incorporated under general laws. He referred in a very pronounced manner to the inefficiency of the existing general law on the subject. The commission to be established as proposed is to be given power to prescribe the course of study to be observed in the institution or any department thereof, and may grant such literary degrees as are usually granted by like institutions and give suitable diplomas.

Under the law of the State there has developed and flourished the National University, with headquarters in the city of Chicago. This institution has not only brought disgrace upon this Commonwealth, but it has discredited American degrees in Europe and Asia, and has been publicly denounced in the British Parliament. It exists on paper and has no standing whatever in any educational institution in the world, and yet it has the insolence to nominate agents to carry on its scandalous traffic in foreign countries, and has scattered its degrees through England, Germany and India for a money compensation.

The dental profession is equally interested in this matter with any other profession. For years past fraudulent degrees have been issued by institutions and sold wherever buyers could be found throughout the world. The extent of the work of this kind is not at all appreciated by the profession, as is now appearing by the work of the commission, appointed by the American Dental Association at its session in 1897.

The most outrageous procedure has been unearthed by this commission and the question now receiving attention is "how efficiently and thoroughly to undo the mischief." It is to be hoped the eyes of the profession will be more and more opened to this iniquitous practice until the whole system shall be clearly exposed to the searchlight of the public, and that the effort for their entire annihilation will not be abandoned till the work is fully accomplished-

